# More Than a Barrier: Exploring the Personal and Professional Impact of Personal Protective Equipment Use in Healthcare

**DOI:** 10.1111/nhs.70231

**Published:** 2025-09-18

**Authors:** Stéphane L. Bouchoucha, Loïc Martin, Emmanuelle Cartron, James J. Lucas, Lim Siew Hoon, Karen McKenna, Ana Hutchinson

**Affiliations:** ^1^ Centre for Quality and Patient Safety Research in the Institute for Health Transformation, Deakin University Geelong Australia; ^2^ Centre for Innovation in Infectious Disease and Immunology Research (CIIDIR), Deakin University Geelong Australia; ^3^ Manipal Academy of Higher Education (MAHE) Manipal College of Nursing (MCON) Manipal India; ^4^ Unité de Formation et de Recherche en Santé, Laboratoire Centre Interdisciplinaire de Recherche Normand en Éducation et Formation (CIRNEF) Unité de Recherche 7454, Université Rouen Normandie Caen France; ^5^ Université Paris Cité, INSERM, UMR 1123—ECEVE Paris France; ^6^ School of Health and Social Development, Deakin University Geelong Australia; ^7^ Institute for Health Transformation, Deakin University Geelong Australia; ^8^ Division of Nursing Singapore General Hospital Singapore; ^9^ Centre for Quality and Patient Safety Research—Epworth HealthCare Partnership, Deakin University Geelong Australia

**Keywords:** adverse reactions, COVID‐19, healthcare workers, masks, pandemic, personal protective equipment

## Abstract

Personal protective equipment used to provide protection to healthcare workers during the pandemic was impacted by shortages in supply and side effects from use. The aim of this study was to explore the prevalence of personal protective equipment (PPE) side effects experienced by healthcare workers in France, including access to, use of PPE, and PPE training. A cross‐sectional descriptive survey was used. Reporting followed the STROBE statement. Three hundred and forty‐six participants completed the survey. Most were female (41.2%), nurses (59.7%) and aged between 31 and 45 years old. Surgical masks were associated with side effects (54.6%), including acne (24.05%), pressure injuries (26.49%) and burning/pain (7.62%). N95 respirator use was associated with side effects (30.43%), of acne (11.88%) and abrasions (11.88%). Side effects were most frequently reported by female participants and those working in COVID‐19 areas. The side effects caused by PPE use and the impact this has on healthcare workers cannot be underestimated. Findings in this study identify the need to develop preventative measures to reduce side effects and improve patient interactions.

**Summary**

- Prolonged use of personal protective equipment increased physical discomfort and side effects for healthcare workers.
- Increased use of personal protective equipment hindered communication and disrupted patient care.
- Despite side effects, most healthcare workers felt protected by using personal protective equipment.

## 1 Background

Controlling the transmission of infectious diseases in healthcare settings is essential for health workers and patients' safety. The emergence of HIV/AIDS in the 1980s saw the widespread adoption of Universal then Standard Precautions (Garner and Simmons 2005) where a focus was placed on the use of personal protective equipment (PPE). Further, the transmission of airborne infections is easily prevented by the use of well‐fitting P2/N95 masks (Howard et al. 2021; Kim and Han 2022); such masks have been widely used to control the spread of tuberculosis or measles and more recently when outbreaks of novel airborne infections such as SARS/MERS emerged (National Health and Medical Research Council 2024; Sarkar 2025).

Personal protective equipment were also widely recommended following the emergence of the Coronavirus Disease 2019 (COVID‐19), caused by severe acute respiratory syndrome coronavirus 2 (SARS‐CoV‐2), first reported in December 2019. Given the airborne nature of the virus (Greenhalgh et al. 2021), the use of PPE was an essential protective measure to prevent transmission of COVID‐19, to maintain the health and safety of the health care workforce ensuring health care service provision remained sustainable despite surges in demand, and to reduce COVID‐19 outbreaks within health care settings (Hoernke et al. 2021; Manookian et al. 2022). Healthcare workers (HCW) were required, often for prolonged time periods, to wear personal protective equipment (PPE). Airborne transmission was not established at the beginning of the pandemic (Zhang et al. 2020) and fast changing guidelines and recommendations resulted in confusion among HCW (Dempster et al. 2022; McKenna et al. 2023) as to which PPE combination would offer the optimal protection against a novel virus with initially little treatment options and/or vaccination. The World Health Organization recommended N95 respirators or surgical masks, goggles or face visors, gloves, aprons and gowns as the necessary PPE and alcohol‐based disinfectants and soap were recommended for hand hygiene (Cordero Jr 2023). The emergence of this novel virus also caused a paradigm shift for HCW and especially nurses: they were required to wear PPE for extended periods of time rather than just when providing care to patients on transmission based precautions (Aloweni et al. 2022; Edward et al. 2024). The extended time that HCWs needed to use PPE when providing care during the COVID‐19 pandemic raised concerns about the increasing incidence of occupationally induced adverse effects (Aloweni et al. 2022). The appearance of such side‐effects, especially when HCW have pre‐existing skin conditions, saw the adoption of ad hoc solutions to protect against side‐effects associated with use of masks and/or other devices (Buranasudja et al. 2021; Vordos et al. 2020). Supply chain issues at the beginning of the pandemic, resulted in PPE shortages, especially N95/P2 masks, and precipitated the use of hand‐made PPE or PPE designed to protect tradesman from inhaling particulates during their work (P1 masks) rather than for protection against inhaling viral fragments such as P2/N95 (Edward et al. 2024; World Health Organization 2021). Side‐effects of masks wearing were described via social media and media from pandemic onset, with pictures depicting nasal and cheeks pressure sores (Solmos 2023). Other side effects such as dehydration and headaches were also described (Dempster et al. 2023; Edward et al. 2024).

One of the main challenges when using PPE is to ensure its efficacy in protecting HCW from pathogens contamination. In France and all other European Union member states, PPE quality is governed by regulation EU 2016/425 (European Union Parliament and Council of the European Union 2016) and PPE manufacturers are required to meet the essential requirements of the regulation. Each PPE item should come with a label guaranteeing adherence to EU regulations. Healthcare facilities and purchasers are responsible for ensuring that PPE purchased complies with this requirement (European Union Parliament and Council of the European Union 2016). In times of shortage, anecdotal evidence showed that some individuals might have used makeshift PPE where quality and protective properties could not be confirmed.

Side effects and PPE shortages both also have the potential to reduce HCW adherence to the use of effective, well‐fitting PPE, thereby increasing their risk of contracting infection and potentially increasing the risk of dermatological side effects (Battista et al. 2021). Although there are some studies that have looked at the impact of PPE side effects and supply chain shortages on HCW (Aloweni et al. 2022), there are no identified studies conducted in France that focus on exploring French HCW experiences.

The health workforce in France comprised a majority of female workers (78%), with only 22% of the workforce working part‐time (French Governement 2024). COVID‐19 was first reported on 14 February 2020 (Tournebize 2022). Two weeks later, the first public health restrictions were introduced, with large gatherings banned and recommendations to use masks and hydroalcoholic hand rubs, and on December 3, 2019, a national strategy linked to vaccination was announced (Jarnoux 2020). Mask wearing in healthcare facilities remained mandatory until 1 August 2022. During the data collection phase, the wearing of P2/N95 masks was still mandatory in healthcare settings. The use of other PPE was advised, as per standard and transmission‐based precautions (Martinot 2025).

The first few months of the COVID‐19 pandemic were marked by a shortage of PPE, including masks. At the start of the pandemic, the state only had a stock of 117 million surgical masks for adults and no strategic state stock of FFP2 masks. As a result, within healthcare facilities, ‘homemade’ masks were occasionally offered, and in some places, bin liner bags were used as gowns to make up for this shortage (Gouvernement Français 2020). The use of improvised PPE during this period played a role in providing minimal, though less than ideal, protection for HCWs and underscored the need for urgent preparedness strategies and improved supply chains. Over subsequent months, various strategies were proposed by the French Ministry of Health, depending on the healthcare services and the patients being treated, through guidelines such as recommendations for the use of face masks (Gouvernement Français 2020).

## 2 Rationale for the Study

Both poor adherence to PPE guidelines and the occurrence of side effects have the potential to increase HCW risk of contracting an infection. In this study, French HCWs experience of PPE use in clinical practice, including perceptions of effectiveness, the impacts of PPE‐related side effects, PPE supply and access, and PPE‐related training needs, were explored. The study findings can be used to inform targeted interventions to improve adherence to PPE guidelines and infection control practices, to better support HCWs providing essential care and treatment, and to improve infection prevention and control preparedness for future pandemics.

## 3 Aims

The aims in this study were to examine the prevalence and impact of PPE‐related side effects among French HCW that provided care during the COVID‐19 pandemic, and to explore their perceptions of PPE effectiveness, supply, access, and training using a cross‐sectional descriptive survey design. To achieve this aim, we used the following research questions:

- What is the prevalence of HCW self‐reported PPE‐related side effects including: (a) frequency, duration, and type of PPE used, (b) type and characteristics of PPE‐related side effects, (c) hand hygiene side effects, and (d) perceptions that PPE use interfered with patient care provision?
- What are HCW's perceptions of access to PPE supplies, their potential risk of COVID‐19 exposure and infection, and the adequacy of the guidance and training they received in appropriate PPE use?

## 4 Materials and Methods

### 4.1 Participants

A convenience sampling approach was used to recruit HCWs working in hospitals in France. The survey was distributed by e‐mail through three professional associations Comité d'entente des formations infirmières et cadres (CEFIEC), Association nationale des directeurs des écoles paramédicales (ANdEP), Association française des directeurs des soins (AFDS), via the professional social network LinkedIn, and finally directly to the network of the two French researchers through snowballing sampling. Any HCW employed during the pandemic was eligible to participate if they had contact with patients/clients. In this study, eligible HCWs were defined as nurses, medical doctors, pharmacists, radiographers, healthcare assistants, physiotherapists, and healthcare students. Non‐HCWs were excluded from participating in the study. Data were collected between 1st September and 18th December 2021. The survey was administered using the online survey platform Qualtrics. As the survey was distributed via email to professional organizations and snowballing, it is not possible to determine how many hospitals received the survey.

### 4.2 Survey Instrument

The survey instrument developed by Aloweni et al. (2022) was used as there were no French instruments. Aloweni et al. (2022) described the development of the instrument, and the questionnaire was also used in Australia (McKenna et al. 2024). For this study, we administered a French version of this questionnaire. There are no methods considered the best for questionnaire translation and cultural adaptation (Epstein et al. 2015). As such, we utilized a method customized for this study. Two native French researchers fluent in English translated the questionnaire. Another two independent French native speakers checked the translations and adjusted these to improve fluency and readability. Finally, one bilingual speaker of English and French translated the questionnaire back into English again. Differences between the back translation and the original were resolved unanimously. Whenever necessary, translations were adjusted to capture contextual and cultural meanings. Face validity was tested through administering the survey instrument to a small sample of nursing academics. No changes were made following this expert review.

### 4.3 Survey Analysis

Data were exported from Qualtrics into SPSS version 26.0 (SPSS Inc.) for analysis. Descriptive statistics, including frequencies and percentages, were used to summarize demographic characteristics, occupation, work location, existence of pre‐existing skin conditions, PPE‐related side effects, and self‐care measures used. Descriptive analyses were also used to summarize participants' perceptions of the impact of side effects on their daily work and patient care, the accessibility of PPE supplies, the likelihood of exposure to infection, and the training provided. Chi‐square tests were used to determine the relationships between categorical variables such as age, gender, occupation, pre‐existing conditions, and level of COVID‐19 risk in the work area (high or low) and PPE‐associated side effects. The level of significance was set at *p* < 0.05. Alongside quantitative and Likert scale responses, we collected free‐text responses to enable participants to expand on some of their responses. Open‐ended responses were analyzed using descriptive content analysis, allowing for the identification of recurring themes and concepts across participants. Short textual answers were coded manually, and similar responses were grouped into broader categories to summarize the main viewpoints (Elo and Kyngäs 2008).

### 4.4 Ethics

This study was conducted in compliance with all stipulations outlined in the protocol. As this study evaluated the way in which healthcare professionals practice, ethical approval in France was not required as per the governing clinical research statement, in accordance with Article R1121‐1 of the French Public Health Code.

Participation in the study was anonymous and voluntary. Informed consent was implied by participants submitting the survey after completion. Participants were informed that as only aggregated data would be reported and that no identifiable data were collected, they could withdraw from the study at any time, but their data could not be removed.

## 5 Results

As the survey was distributed electronically with participants accessing the online survey using a QR Code or an email link, it was not possible to determine the total number of participants who were invited to participate in the study. Response rates were calculated as the total number of completed surveys divided by the total number of surveys accessed by potential participants.

A total of 346 participants completed the survey (Table 1). Most respondents were female (41.2%) and aged between 31 and 45 years old, and 59.7% were registered nurses, followed by clinical managers/directors (9.9%), healthcare assistants (9.3%) and allied health (6.7%). Sixty‐three respondents (18.3%) reported a pre‐existing skin condition, with dry skin reported most frequently (28.8%) followed by atopic dermatitis (19%) and allergic dermatitis (9.5%). Most respondents (90.7%) were employed full‐time, with 70.9% of respondents working in dedicated COVID‐19 areas and 40.3% in acute care areas. Table 1 provides more details on work locations.

**TABLE 1:** Participants demographics.

	*N* = 346	Dedicated COVID areas	Non Dedicated COVID areas
		*N* = 251	*N* = 95
	*n* (%)	*n* (%)	*n* (%)
Gender			
Female	266 (77.1)	206 (82.1)	60 (63.2)
Male	72 (20.1)	42 (16.7)	30 (31.6)
Non‐binary	0	0	0
Not specified	8 (2)	3 (1.2)	5 (5.3)
Age			
< 30	57 (16.5)	43 (21.1)	14 (14.8)
31–45	142 (41.2)	105 (41.8)	37 (39.0)
46–60	136 (39.4)	98 (39.0)	38 (40)
≥ 61	8 (2.3)	5 (2)	3 (3.2)
Missing	3 (0.9)	0 (1.2)	3 (3.2)
Occupation			
Medical	12 (3.5)	8 (3.2)	4 (4.2)
Registered nurse	206 (59.7)	150 (59.7)	56 (58.9)
Advanced practice nurse	15 (4.3)	9 (3.6)	6 (6.3)
Clinical managers/Directors	34 (9.9)	26 (10.3)	8 (8.4)
Students	21 (6.1)	16 (6.4)	5 (5.2)
Allied health	23 (6.7)	21 (8.4)	2 (2.1)
Healthcare assistants	32 (9.3)	20 (8)	12 (12.6)
Other	3 (0.5)	1 (0.4)	2 (2.1)
Employment status			
Full time	313 (90.7)	227 (90.4)	86 (90.5)
Part time	26 (7.5)	21 (8.4)	5 (5.3)
Casual	7 (2)	3 (1.2)	4 (4.3)
COVID‐19 risk setting			
Dedicated COVID area	251 (79.5)		
Non dedicated COVID area	95 (18.8)		

Primary work location	Total	Dedicated COVID areas	Non‐Dedicated COVID areas
	*N* = 346	*N* = 251	*N* = 95
	*n* (%)	*n* (%)	*n* (%)
Acute care	119 (34.4)	87 (34.6)	32 (33.7)
Intensive care units	51 (14.7)	18 (7.2)	33 (34.7)
Rehabilitation	41 (11.8)	33 (13.1)	8 (8.4)
Leadership and management	33 (9.5)	27 (10.7)	6
RACF	26 (7.5)	23 (9.2)	3 (3.2)
Mental health	21 (6.1)	21 (8.4)	0
COVID screening	19 (5.5)	11 (4.4)	8 (8.4)
Education	13 (3.7)	11 (4.4)	2 (2.1)
Radiology	12 (3.5)	12 (4.8)	0
Pediatrics	4 (1.1)	4 (1.6)	0
Operating room	4 (1.1)	4 (1.6)	0
Pharmacy	3 (0.9)	0	3 (3.3)
Pre‐existing skin conditions			
Yes	63 (18.3)	43 (17.1)	20 (21.0)
Skin conditions^a^			
Atopic dermatitis	17 (27.2)	14 (32.5)	3 (15)
Allergic dermatitis	31 (49.6)	21 (49.9)	10 (50)
Dermatosis	12 (19.2)	9 (21)	3 (15)
Psoriasis	6 (9.6)	4 (9.3)	2 (10)
Acne	3 (4.8)	2 (4.6)	1 (5)
Urticaria	1 (0.6)	1 (2.3)	0
Others	5 (8)	3 (6.9)	2 (10)

*Note:* Others included: zona, scabies, and sun dermatitis.

^a^ Adds up to over 100% as some respondents selected multiple responses.

### 5.1 Types of PPE Used

When respondents were asked about their PPE use during the pandemic (Table 2), 26.6% (*n* = 92) reported using a surgical mask only, 69.4% (*n* = 240) reported wearing an N95/P2 respirator. Eye protection was used by most respondents, with goggles (*n* = 234; 67.9%) being used more than face shields (*n* = 129, 37.3%).

**TABLE 2:** Types of PPE used.

Types of PPE used	*N* = 346; *n* (%)
No masks	5 (1.4)
Surgical masks (only)	92 (26.6)
FFP1	9 (2.6)
N95/FFP2 respirator	240 (69.4)
Eye protection	246 (100)
Goggles	234 (67.6)
Face shields	129 (37.3)
Apron	201 (58.1)
Waterproof long sleeve gown	93 (26.9)
Fabric gown	207 (58.8)
Single gloves	275 (79.5)
Double gloves	41 (11.9)
Shoe covers	81 (23.4)
Hair covers	248 (71.7)
No PPE (except gloves)	47 (13.6)
Other	9 (2.6)

*Note:* Respondents could choose several options, thus adding to over 100% in certain categories.

Abbreviation: PPE, personal protective equipment.

### 5.2 Reported Side Effects From PPE Use

Reported side effects experienced from PPE use are shown in Table 3. Surgical masks were commonly associated (*N* = 189, 54.6%) with side effects such as: acne (24.05%), pressure injuries (26.49%) and burning/pain (7.62%) on the cheeks (24.8%), nose (18.2%) and behind the ear (38.1%). The high reported frequency of side effects associated with surgical mask use reflects that the majority of participants used this type of PPE.

**TABLE 3:** Frequency of reported side effects by personal protective equipment type (*N* = 346).

	Goggles *N* (%)	Face shield (19/129)	Surgical mask (189/341)	N95 respirator (149)
Skin related				
Skin tear	—	—	—	—
Blister	—	—	—	4 (1.16)
Acne	4 (12.90)		82 (24.05)	41 (11.88)
Abrasion	5 (16.13)	1 (0.77)	38 (11.14)	41 (11.88)
Eczema	2 (6.45)	—	11 (3.23)	9 (2.61)
Allergic reaction	1 (3.22)	—	26 (7.62)	12 (3.48)
Dry skin	—	—	2 (0.59)	—
Pressure related				
Burning/pain	4 (12.90)	2 (1.55)	26 (7.62)	27 (7.83)
Pressure injuries	15 (48.39)	8 (6.20)	90 (26.39)	105 (30.43)
Headache/migraine	1 (3.22)	2 (1.55)	4 (1.17)	4 (1.16)
Vision				
Blurred vision	1 (3.22)	2 (1.55)	—	1 (0.30)
Impaired vision	—	—	—	—
Fogging	7 (22.58)	5 (3.87)	2 (0.59)	2 (0.58)
Hearing				
Impaired hearing	—	1 (0.77)	—	—
Respiratory				
Asthma	—	—	—	1 (0.30)
Shortness of breath	—	—	6 (1.76)	6 (1.74)
Respiratory—other	—	—	—	2 (0.58)
Others	1 (3.22)	—	28 (8.21)	5 (1.45)

*Note:* Participants could report side effects associated with multiple types of PPE; other included signs of dehydration, dry mouth, cough.

**FIGURE 1:**
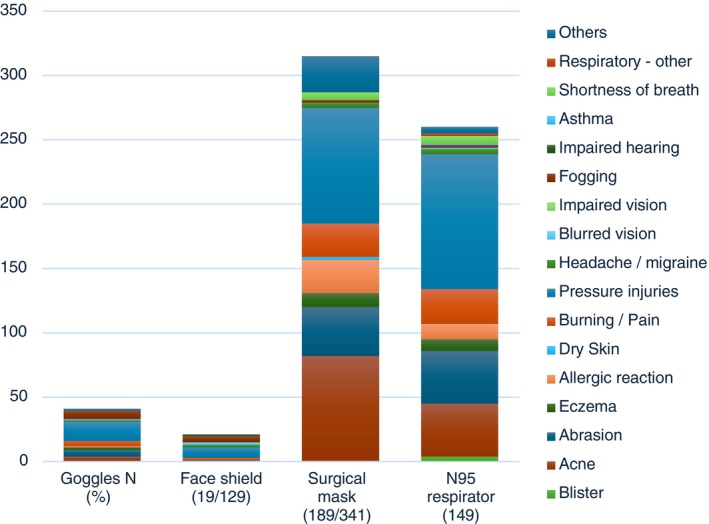
Frequency of reported side effects by PPE type (*N* = 346). Note. Participants could report side effects associated with multiple types of PPE; other included signs of dehydration, dry mouth, cough.

In relation to N95 respirator use, 43.1% reported an adverse effect, with pressure injuries the most common (30.43%) followed by acne (11.88%) and abrasions (11.88%). The location of side effects was similar to those reported with surgical masks, with the nose (27.5%), cheeks (19.4%) and behind the ear (26.5%) the most frequently reported. Side effects were reported to have affected professional activities for 11.3% (39) of respondents, with no one reporting having used skin protection/measures to prevent side effects. Further details are given in Figure 1/Table 3.

### 5.3 Factors Impacting the Frequency of Reported Side‐Effects

Overall, there were no associations between the number of hours worked and reported side effects; this is probably due to the majority of respondents reporting being in full‐time employment. People working in COVID‐19 designated areas were more likely to wear an N95 respirator than those working in other areas of the health service (*χ* ^2^ (1, *N* = 345) = 11.38, *p* = 0.003).

### 5.4 Side Effects of Wearing an N95 Respirator by Work Locations and Gender

A chi‐square test of independence indicated a significant association between reporting side effects from wearing an N95 respirator by work location, with more side effects reported from people working in a designated COVID‐19 area, *χ* ^2^ (1, *N* = 346) = 20.22, *p* = 0.001. When considering the association between reporting side effects and gender, women were more likely to have reported such side effects, *χ* ^2^ (1, *N* = 346) = 54.37, *p* < 0.001. An exploration of the association between reporting side effects by work location and by gender revealed no significant associations: (Women: *χ* ^2^ (2, *N* = 204) = 5.65, *p* = 0.06; Men: *χ* ^2^ (2, *N* = 64) = 1.63, *p* = 0.44; Total: *χ* ^2^ (2, *N* = 268) = 4.15, *p* = 0.12).

### 5.5 Hand Hygiene Practices

**FIGURE 2:**
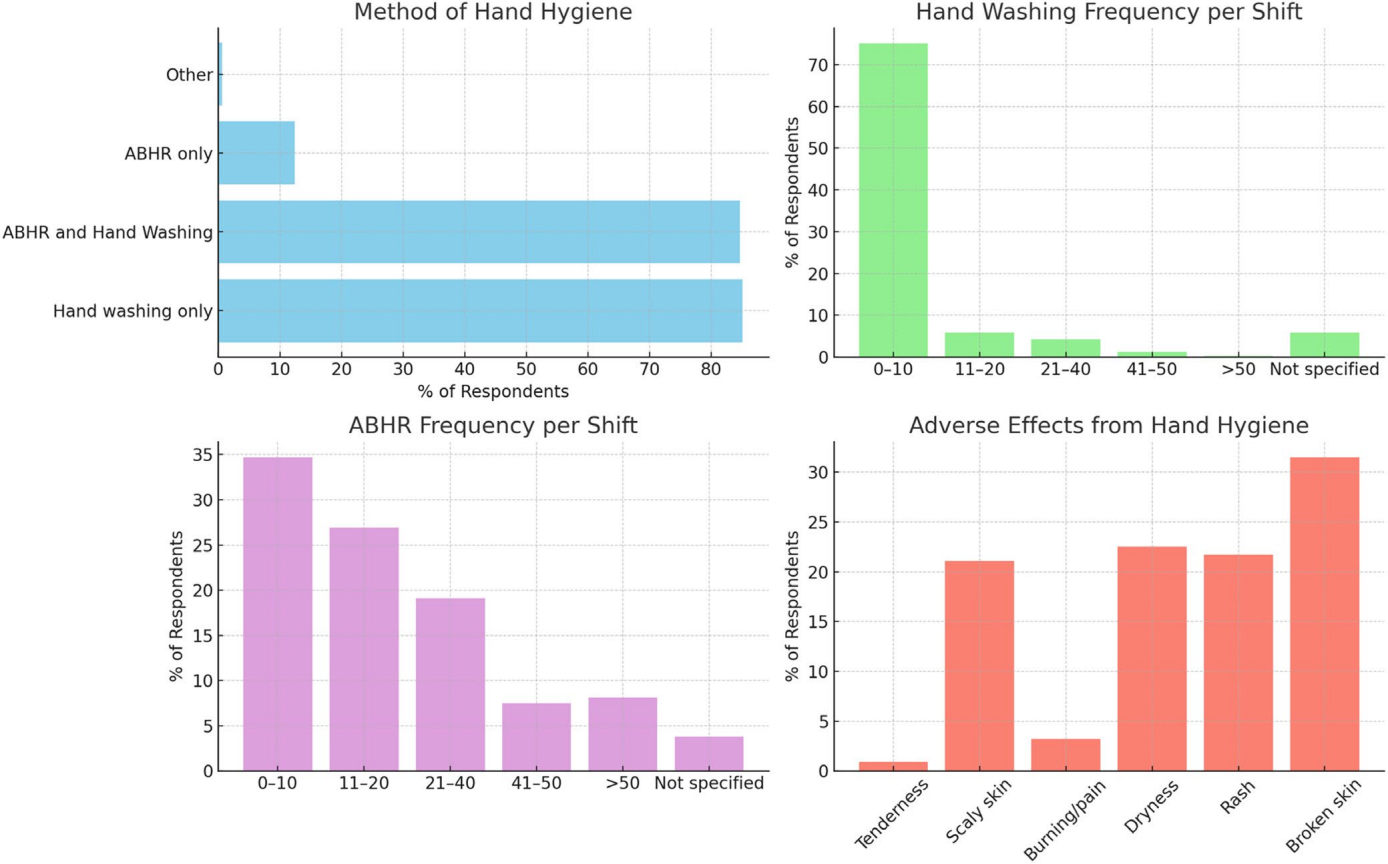
Hand hygiene practices during COVID‐19. Note: ABHR, alcohol based hand rub.

Over half (191, 55.2%) of respondents reported an adverse reaction to hand hygiene products (Figure 2). The most reported were broken skin (109, 31.5%), followed by dry skin (21.7%), rash (21.7) and scaly skin (21.1). Skin reaction severity was reported as varying between very slight and severe. Over 49% (171) of respondents stated that they used measures to prevent the occurrence of hand hygiene‐related side effects, with the use of hydration cream 100% (171), change of soap type (12%) and an increase in drying time/careful attention to drying hands (3.3%; *n* = 11).

### 5.6 Impact of PPE on Patients/Healthcare Workers Relationship

In free‐text responses, most respondents felt that wearing PPE modified the therapeutic relationship and/or made looking after patients uncomfortable. Participants stated that PPE use made care provision less inclusive (i.e., more difficult for people relying on lip reading and/or less able to hear); made non‐verbal communication more difficult, and that especially at the beginning of the pandemic some patients expressed increased anxiety that could create a barrier to care.

### 5.7 Confidence in PPE Availability and Protection Provided

Respondents were also asked whether they were confident in the protection afforded by N95 respirators, and the majority (268; 77.4%) were confident or strongly confident of being protected. When asked whether they thought that some care activities increased their risks of being exposed to COVID‐19, 53.5% (*N* = 185) were affirmative. Such activities were endotracheal suction, mouth care, intubation, and/or use of aerosol therapy. Despite these perceived risks, only 41.9% (*N* = 145) agreed that there was sufficient PPE to meet departmental needs. A majority, 66.7% (*N* = 229) agreed they had sufficient training in PPE use, and 67.3% (*N* = 233) were confident in their understanding of correct PPE selection. The majority agreed that there were sufficient visual reminders (75.4%, *N* = 261) about correct PPE use and that these were useful (62.5%, *N* = 216). Spotters were available in 42.5% (*N* = 147) of respondents' areas, and in 59% (*N* = 118), respondents stated that having spotters helped with donning and doffing PPE (Table 4). These responses indicate that although there were some implied concerns about PPE supply, most respondents agreed that sufficient supports were available to promote correct PPE use in the clinical practice setting.

**TABLE 4:** Availability of personal protective equipment and protection.

	Strongly agree	Agree	Neither agree or disagree	Somewhat disagree	Strongly disagree	No answer
Wearing PPE modifies patent/healthcare worker relationship	53.8% (186)	26.0% (90)	9% (31)	4.9% (17)	1.7% (6)	4.6% (16)
Wearing a gown makes care giving more difficult	9.8% (34)	14.7% (51)	18.8% (65)	28.3% (98)	17.1% (59)	11.2% (39)
Wearing PPE makes looking after a patient uncomfortable	36.1% (125)	35.5% (123)	9.2% (32)	6.4% (22)	2% (7)	10.7% (37)
The recommended PPE are readily available in my department whenever they are needed	15% (52)	27.2% (94)	13.9% (48)	25.1% (87)	15.9% (55)	2.9% (10)
There is enough PPE supply for all healthcare staff in my department	15.3% (53)	26.6% (92)	13.3% (46)	22.5% (78)	19.4% (67)	2.9% (10)
I have had sufficient training in the correct use of PPE	29.5% (102)	36.7% (127)	12.7% (44)	10.7% (37)	5.8% (20)	4.6% (16)
I have a clear understanding of the indications for use of different types of PPE	26.3% (91)	41% (142)	15.6% (54)	10.1%% (35)	3.8% (13)	3.1% (11)
There are sufficient visual reminders to remind on the use of PPE	29.2% (101)	46.2% (160)	10.4% (36)	7.2% (25)	2.9% (10)	4.0% (14)
The visual reminders on the following of PPE are a useful reminder to me	24.3% (84)	38.2% (132)	21.1% (73)	5.8% (20)	2.6% (9)	7.1% (28)

## 6 Discussion

This study aimed to understand the prevalence of PPE side effects experienced by HCWs in France, and to evaluate the HCWs perception of access to and use of PPE. To our knowledge, this is the first study to evaluate the use of PPE and the potential related side effects in a population of French HCWs.

This study reported similar findings to comparative studies from different countries, with the majority of the respondents being female HCWs, predominantly nurses, aged between 30 and 40 (Galanis et al. 2021). Reports of pre‐existing conditions were lower in this study, with 18.3% reporting pre‐existing skin conditions, compared to comparison studies with 47.6% of participants reporting side effects in Australia (McKenna et al. 2024), and 45.5% in Singapore Aloweni et al. (2022). Dry skin (28.8%) followed by atopic dermatitis (19%) were the most frequently reported side effects in all three studies, with similar rates reported in this and the Australian study. The Singapore study findings were that 58% of participants reported dry skin, and 34.2% reported atopic dermatitis.

In this study 26.6% of participants reported wearing only a surgical mask, and 69.4% wearing a N95 respirator, despite this more side effects were experienced from surgical masks (54.6%) compared to N95 respirator use (43.1%). Kaur et al. (2024) reported up to 96% of side‐effects for N95 masks. The difference with our report may be because they only surveyed HCWs working in areas where patients with COVID‐19 were looked after while our study included a wider range of settings. Side effects from surgical masks in the current study were reported on the cheeks (24.8%), nose (18.2%) and behind the ears (38.1%). N95 respirator related adverse effects were reported as pressure injuries (43.1%), followed by skin related issues (11.8%). The location of injuries from N95 respirators was similar to surgical mask use affecting the nose (27.5%), behind the ear (26.5%), and cheeks (19.4%). This is consistent with the tight face seal required for effective PPE and is representative of the most commonly reported injury locations in the literature (Abiakam et al. 2021); Kaur et al. (2024). Causative factors could be linked to the design and materials used in N95 respirators, with most devices taking a one size fits all approach, designed for the white male face shape, using stiff materials that are difficult to conform to different face shapes (Abiakam et al. 2021). The finding that surgical masks have a high incidence of side‐effect is important to consider. Outside of pandemic conditions, surgical masks are widely used and considering these side effects is essential when trying to enhance workforce wellbeing.

Due to global PPE shortages, extended use of PPE for longer time periods was implemented as a strategy to ensure HCWs were protected from transmission risks and to preserve PPE supply (Boškoski et al. 2020; Burki 2020). Future pandemic planning should consider potential supply chain issues as these issues might increase the risk of healthcare workers being infected by pathogens while on duty.

In this study there was no identified association between the number of hours worked and reported side effects from PPE used. Similar studies identified an increase in reported rates of PPE reactions associated with an increased duration of time spent in PPE (Galanis et al. 2021). Additional contributing risk factors associated with the extended use of PPE have been identified as higher consecutive days with PPE used, higher grades of PPE used, and greater exposure to patients with COVID‐19 infection requiring more PPE use (Chavan et al. 2024; Galanis et al. 2021).

Consistent with other studies, this study identified that females were more likely to report PPE‐related side effects (Aloweni et al. 2022; McKenna et al. 2024; Zarei et al. 2024). Differences in hormones, genetics, and skin care behaviors have been identified as potential causative factors that may explain gender‐based differences in the frequency and severity of PPE side effects (Abiakam et al. 2021; Galanis et al. 2021).

PPE use throughout the pandemic was acknowledged to have an impact on patient care, as it presented challenges in communicating with both patients and colleagues (Dempster et al. 2023, 2024; Galanis et al. 2021). In this study, most respondents reported wearing PPE had an impact on therapeutic relationships with patients, made care provision feel less inclusive, and restricted non‐verbal communication. Similar studies have identified that nurses providing bedside care and spending most of their shifts in PPE have also reported these difficulties in providing patient care, including a slower delivery of care, restricted movements, and exacerbated feelings of exhaustion, irritability, and tiredness (Hoernke et al. 2021; Zarei et al. 2024). Communication problems with elderly patients who rely on lip reading were exacerbated, as were difficulties in building rapport with patients (Dempster et al. 2023, 2024; Edward et al. 2024; Gutiérrez‐Puertas et al. 2023; Hoernke et al. 2021). Despite the negative associations with PPE, most respondents felt confident that they were protected by their PPE (77.4%). Studies undertaken during previous epidemics have identified that HCWs feel protected and confident to deliver care to patients when there are adequate provisions of PPE available for use, and the workforce is trained in the correct use of PPE (Hoernke et al. 2021; Liang et al. 2024).

In this study, over half the respondents (55.2%) reported an adverse reaction to hand hygiene products with preventative measures, including the use of hydration creams and increased drying times, implemented by over 49% of respondents. Increased hand hygiene practices and use of PPE throughout the pandemic have been associated with an increase in reported hand‐related dermatitis through the removal of bacterial flora on the skin, and a breakdown of the protective skin barrier (Barnawi et al. 2021; Galanis et al. 2021; Hagiya et al. 2024).

Results in this study show that the experience of people working in dedicated COVID‐19 areas could be applied to any areas where people must use PPE for an extended period with infectious diseases/pathogens transmitted through the air: for example, measles/other respiratory viruses and/or haemorrhagic fevers. Recent increases in vaccination hesitancy have seen measles outbreaks resurfacing (Mathis 2025) and call for healthcare workers to implement standard and transmission‐based precautions. As seen in the current study, it is essential for organizational leaders to consider the PPE‐related side effects experienced by their workforce and to make adjustments to healthcare workers' work schedules to increase PPE adherence and reduce pathogen transmission risks.

In addition, the findings in this study are applicable for subsequent and potential outbreaks of novel pathogens (particularly when the transmission mode is unclear and maximum protection of the health workforce is required) and/or during ongoing outbreaks of COVID‐19. The results in this study are also essential to consider in future pandemics. The recent COVID‐19 pandemic caused many changes in healthcare sectors and highlighted some challenges such as a lack of adequate planning (Bouchoucha and Havers 2025; Bush et al. 2023). It is therefore essential that we reflect on lessons learned during the COVID‐19 pandemic and implement these for ongoing pandemic planning.

## 7 Strengths and Limitations

This study is the first one to investigate French healthcare workers experience of PPE side effects and perceived impact on patient care. The survey was distributed during a peak time of widespread use of PPE by all HCW providing direct clinical care. The survey tool used in this study was translated following best practice requirements, thereby minimizing potential issues related to accuracy and interpretation of survey items. The survey tool has also been used in studies in Singapore and Australia, thus enabling comparability of data across different contexts.

Despite strengths, there are some limitations that need to be discussed. The main one is the self‐reported aspect of the study. It is possible that the design of the study introduced a selection bias, wherein only people that experienced side effects or who had a strong opinion on PPE use decided to complete the questionnaire. Although supply chain issues were mostly resolved when the study was conducted, shortages of PPE might also have influenced reported outcomes, as there was possible variation in the quantity and type of PPE available to participants.

## 8 Conclusions

While the protective benefits of PPE are evident, the side effects caused by PPE use and the impact of this on HCWs and patient care provision cannot be underestimated. This study has provided a firsthand perspective of experiences with PPE from frontline HCWs in France and contributes to the evidence base for the adverse impacts of PPE use on HCWs and the provision of patient care. Strategies to prevent and minimize PPE side effects, discomfort, improve compliance with use, and improve interactions with patients must be considered and evaluated in further research.

## 9 Relevance for Clinical Practice and Future Research Needs

Experiencing PPE side‐effects can have an impact on HCWs willingness to use PPE and their adherence to transmission‐based precautions. Lack of adherence is problematic as it can increase HCWs risk of being infected by novel pathogens and further increase the risk of cross transmission. As HCWs, particularly nurses, represent most of the health workforce, it is essential to consider the frequency and type of PPE‐related side‐effects they experience, so that effective mitigation strategies can be developed to improve PPE adherence and decrease exposure to pathogens in the work setting. It is also essential to implement strategies that can address the impact of PPE use on teamwork and care delivery, to ensure optimal quality and patient safety is maintained during a crisis (Dempster et al. 2023, 2024).

Although in this study we did not find a statistically significant association between hours worked and side‐effects, individuals working in COVID‐related areas reported more side‐effects. In general, individuals working in COVID‐affected areas would be wearing PPE continuously for the duration of their shift (as there is the potential for exposure in all work areas on the ward). It therefore seems possible that continuous PPE use over a shift may increase the risk of side‐effects and is potentially a more important risk factor than absolute hours worked. This needs to be evaluated prospectively under controlled conditions. If continuous work increases side‐effects, it is paramount to adapt working practices and provide HCWs with planned breaks.

The COVID‐19 pandemic has given PPE manufacturers the opportunity to benefit from observing wide‐scale use of masks. Despite this large scale natural experiment, efforts to redesign and improve PPE have focused on life‐cycle assessments and advancements in materials, manufacturing, and technology, with an emphasis on improving filtration and breathability, antimicrobial coatings, and developing reusable PPE to improve sustainability (Ogbuoji et al. 2021; Zeng et al. 2024). Our findings highlight that addressing side effects experienced by HCW is essential to increasing wearers' wellbeing, which in turn could reduce the likelihood that HCW are exposed to infectious disease threats through poorly fitting masks or side effects leading to non‐adherence to PPE use.

Addressing side effects is twofold, through better attention to occupational health and safety and through engaging with manufacturers. Designing education programs that consider reported side effects is key to increasing wearability, workers safety, and comfort. These programs need to encompass information on the need for breaks to alleviate dehydration but also the use of spotters to make sure PPE fit is optimal and risk‐free. On the manufacturing side, advancements need to address the re‐designing of PPE ergonomics to improve fit and wearability, thus reducing wearer side effects and discomfort. Further research is essential to evaluate optimal PPE design that focuses on developing face masks that provide an optimal seal for individuals with a wide range of facial features to decrease excessive pressure, bruising, or skin abrasion associated with poorly fitted masks. Innovation in the materials used in mask manufacture is critical to ensure protection from pathogens while minimizing diaphoresis and health exhaustion. There is an opportunity to take the lessons from this widescale pandemic and for manufacturers and HCW to work together to address workers comfort while continuing to decrease exposure to airborne infection risks.

## Author Contributions

**Stéphane L. Bouchoucha, Ana Hutchinson, Lim Siew Hoon, Karen McKenna:** conceptualization, methodology, data analysis, writing – original draft preparation. **James J. Lucas:** data analysis, writing – original draft preparation. **Emmanuelle Cartron, Loïc Martin, Karen McKenna:** data curation, writing – original draft preparation. All authors contributed to the writing, reviewing and editing of the manuscript.

## Ethics Statement

Ethical approval was not required for this study as this study evaluated the way in which healthcare professionals practice and as per the law governing clinical research in France, in accordance with Article R1121‐1 of the French Public Health Code, this study was exempt from ethical approval.

## Conflicts of Interest

Stephane Bouchoucha is an Associate Editor for Nursing & Health Sciences and a co‐author on this article. The manuscript was managed by editors unaffiliated with the author or institution and monitored carefully to ensure there is no peer review bias. All other authors declare no conflicts of interest.

## Data Availability

The data that support the findings of this study are available on request from the corresponding author. The data are not publicly available due to privacy or ethical restrictions.
